# Benign and malignant ovarian steroid cell tumors, not otherwise specified: case studies, comparison, and review of the literature

**DOI:** 10.1186/1757-2215-6-53

**Published:** 2013-07-22

**Authors:** Wei Jiang, Xiang Tao, Fang Fang, Shaofen Zhang, Congjian Xu

**Affiliations:** 1 Department of Gynecology, Obstetrics and Gynecology Hospital, Fudan University, 419 Fangxie Road, Shanghai 200011, P. R. China; 2 Department of Pathology, Obstetrics and Gynecology Hospital, Fudan University, Shanghai, People’s Republic of China; 3 Shanghai Key Laboratory of Female Reproductive Endocrine Related Diseases, Shanghai, People’s Republic of China

## Abstract

Ovarian steroid cell tumors, not otherwise specified (NOS) are rare sex cord-stromal tumors of the ovary with malignant potential. So far only a few cases were reported in English literature through the Pubmed search. Here we report two cases of such tumor, one was benign (first case underwent laparoscopic cystectomy) and the other was malignant (died 10 months later after initial diagnosis), both presented with amenorrhea and clinical signs or symptoms of virilization. In malignant case, we provided evidence (tumor embolus) in addition to the reported five characteristics associated with malignancy. On further evaluation, laboratory investigations revealed hyperandrogenism in the male range, while follicle stimulating hormone (FSH) and luteinising hormone (LH) levels were within normal limits. Various aspects of the presentation, diagnosis, and treatment of these tumors are discussed.

## Background

Ovarian steroid cell tumors, not otherwise specified (NOS), are rare sex cord-stromal tumors of the ovary with malignant potential, accounting for less than 0.1% of all ovarian tumors [1]. These tumors should be considered a cause of isosexual precocious puberty in children and virilisation in adults, such as hirsutism, temporal balding, and amenorrhea. In literature only a few cases of steroid cell tumors, NOS, have been described [2-11]. Most of the reported cases only described one benign case. This report describes cases of a benign and a malignant steroid cell tumor (NOS) in two young adult women who presented with amenorrhea and virilization. Aspects of the presentation, diagnosis, and treatment of these tumors are discussed. Prior written consent of the patients for the use of these clinical materials for research purposes, and approval from the Institutional Ethical Board (IRB) in the Obstetrics and Gynecology Hospital of Fudan University were obtained.

## Case presentation

### Case 1

A 23-year-old female came to our hospital in April 2009 with months’ history of increasing facial and truncal hair, acne. The patient had been amenorrheic for 2 years prior to the onset of her virilizing symptoms. In the recent one year, she was treated as Polycystic Ovary Syndrome (PCOS) in the local hospital, but the signs and symptoms were deteriorated.

Physical examination revealed a 56 kg, normotensive female with obvious facial hair and atrophy of the breasts. Excessive hair was present on her lower abdomen and thighs. Pelvic examination was notable for an enlarged clitoris and a 5 cm right adnexal mass. Abdominal ultrasound identified a 64 × 52 × 51 mm, solid, left ovarian mass. Doppler evaluation of intratumoral blood vessels confirmed a low resistance to flow. No ascites or other abnormalities were present. A CT scan of the pelvis confirmed the ultrasound findings and detected no adrenal gland enlargement or tumor. Laboratory analysis revealed normal values of folicle stimulating hormone (FSH), luteinising hormone (LH), serum prolactin (PRL) and cortisol. Total serum testosterone was 3.68 ng/ml (normal 0.15-0.51 ng/ml), serum dihydroepiandosterone sulfate (DHEA-S) was 403.1 μg/dl (normal 19-391 μg/dl) and serum Sex hormone-binding globulin (SHBG) was 21.16 nmol/l (normal 24-230 nmol/l). (Table 1) The preoperative diagnosis was testosterone-producing sex-cord stromal cell tumor.

**Table 1:** Reproductive endocrine hormone levels before and after surgery of the two patients

	**FSH (U/L)**	**LH (U/L)**	**E2 (pg/ml)**	**PRL (ng/ml)**	**T (ng/ml)**	**DHEA-S (μg/dl)**	**SHBG (nmol/L)**	**Cortisol (μg/dl)**
Normal values of age	5.2-14.4	1.8-7.4	18-195	3.5-24.2	0.15-0.51	19-391	24-230	4.3-22.4
Case 1								
Before surgery	6.2	4.5	57	21.84	3.68	403.1	21.16	22.59
1 week after surgery	7.6	5.2	20	10.05	0.48	146.6	34.21	18.36
1 months after surgery	6.5	3.3	19	13.98	0.54	220.8	45.46	12.17
1 year after surgery	6.8	6.7	49	7.67	0.49	323.5	38.22	14.47
Case 2								
Before surgery	5.4	4.7	23	12.12	13.16	736.11	18.34	19.67
1 week after surgery	6.2	7.3	38	15.32	2.77	512.2	20.12	12.46
1 months after surgery	5.5	6.1	26	18.11	6.11	544.3	18.42	11.31
3 months after surgery	4.8	4.5	22	16.35	8.22	580.2	21.27	14.79

**Figure 1:**
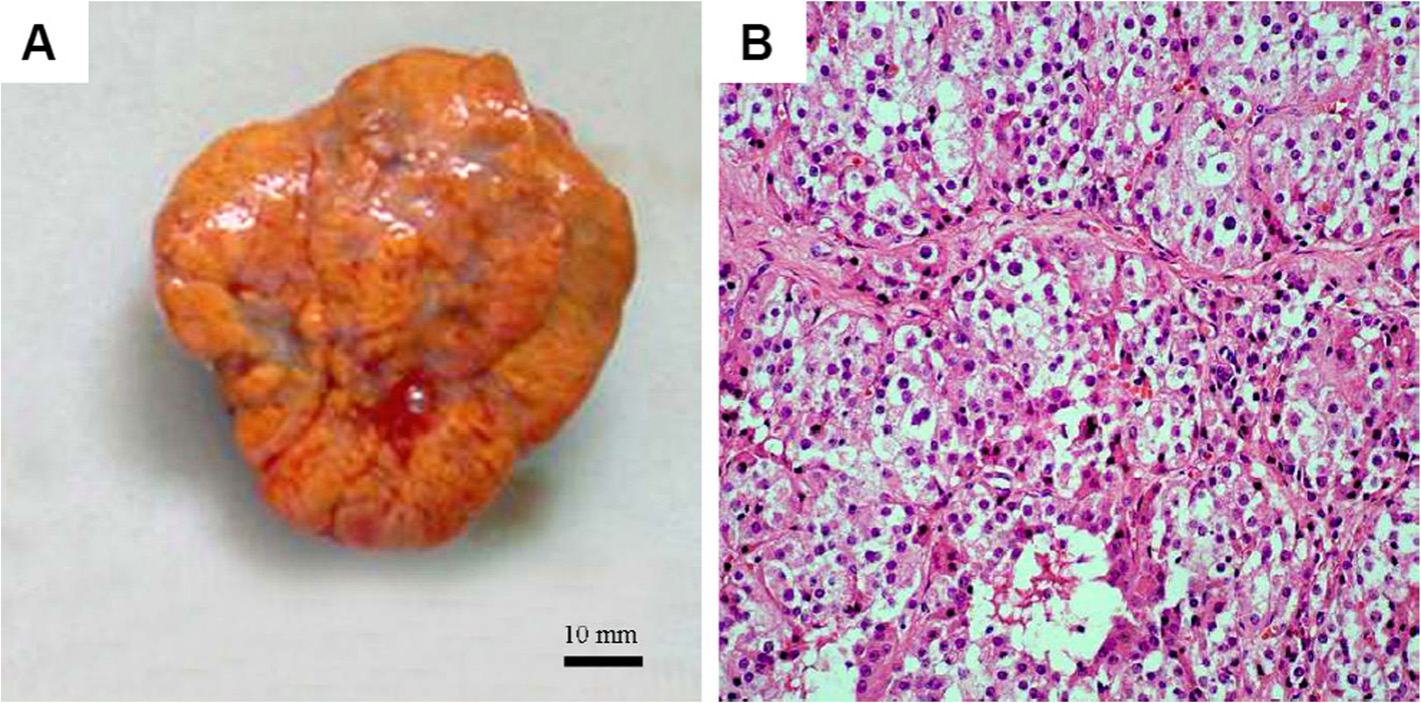
**Benign ovarian steroid cell tumor, NOS. (A)** Macroscopic appearance: Note the well-circumscribed, solid, yellow-brown tumor. **(B)** Microscopic appearance: cells with small round nuclei, mild atypia, and no mitosis are arranged in a diffuse pattern of columns or nests separated by a rich vascular network. The cells demonstrate abundant pale cytoplasm and no crystals of Reinke (H&E, 20×).

With sufficient preparation, the patient underwent laparoscopic examination which identified a 6 × 5 × 5 cm, enlarged right ovary. A well-circumscribed, yellow-white, solid mass was detected and removed by cystectomy. Frozen section of the right ovary mass demonstrated a benign ovarian stromal tumor. Peritoneal washing was negative. Microscopically, tumor cells were arranged in a diffuse pattern or columns or in nests separated by a rich vascular network. The majority of cells had small round nuclei, an abundant amount of vacuolated cytoplasm, mild atypia, and no significant necrosis or mitotic activity. No crystals of Reinke were identified. (Figure 1) Immunohistochemistry revealed a result of CKpan (-), Vimentin (-), Epithelial membrane antigen (EMA) (-), ER (+), PR (++), CD99 (++), Calretinin (++), Inhibin-α (+++), ki-67 (2%+), P53 (-). These findings were consistent with a benign ovarian steroid cell tumor, NOS.

**Figure 2:**
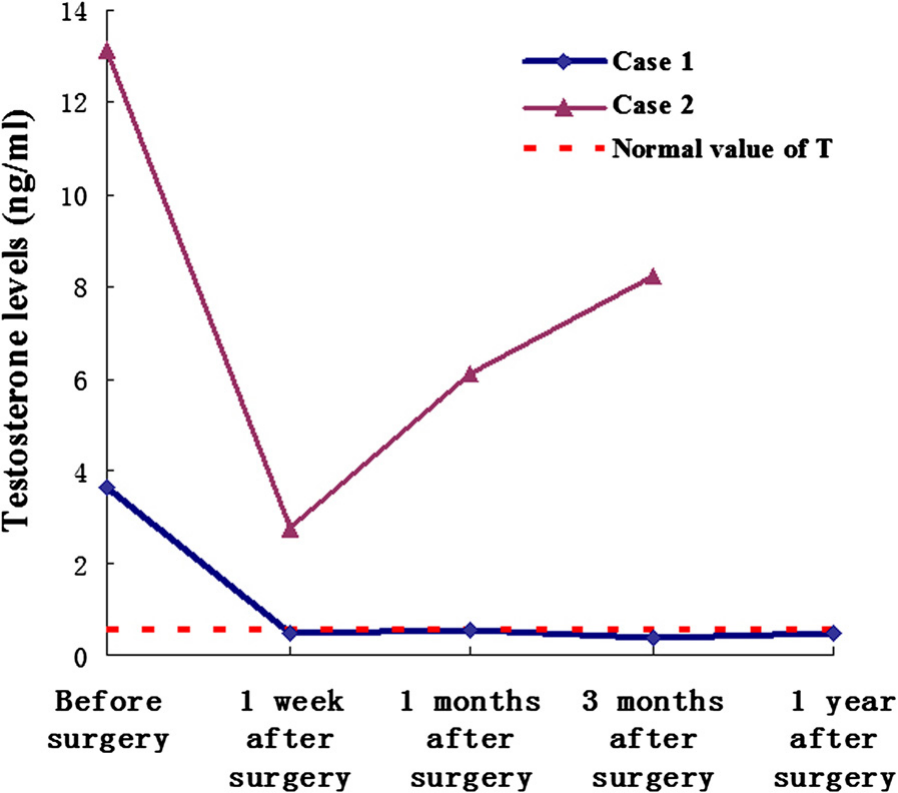
The dynamic changes of the patients’ serum testosterone level before or after surgery.

Following an uneventful recovery, the patient’s serum total testosterone has been within normal limits 7 days after surgery (Figure 2 and Table 1). Two months after the surgical excision, her facial hairs had disappeared, her periods had normalized to a regular 4/30-day cycle, and she had a normal feminine voice. She is alive with normal cycle and without any evidence of disease 3 years after diagnosis of ovarian steroid cell tumor, NOS.

### Case 2

This 21-year-old unmarried woman was referred to our hospital in May 2007 because of a 7-year secondary amenorrhea history and a pelvic mass. She attained menarche at the age of 12 years. Amenorrhea occurred when she was 14 years old, accompany with increasing hirsutism, acne, deepening voice, and acanthosis nigricans-like change of skin. She came to a local hospital in 2003. Her serum total testosterone level was 5.38 μg/L. An abdominal ultrasound scan detected a 60 × 55 × 52 mm, solid, right ovarian mass. Laparotomy was suggested by her local doctors, while the patient refused receiving any further treatment.

After 4 years, in May 2007, she came to our hospital with severe hirsutism, acanthosis nigricans, and acne. Physical examination revealed a big palpable mass in the lower abdomen. A transvaginal ultrasound scan of the pelvis demonstrated moderate amount of ascites and heterogeneous masses in both ovaries, with the size of 190 × 136 × 158 mm in left and 144 × 108 × 90 mm in right, respectively. The uterus was normal in size. The presumptive diagnosis of malignant ovarian tumor was made. The serum total testosterone rise to 13.16 μg/L, while the FSH, LH, E2, PRL, and cortisol levels were within the normal range (Table 1).

The patient was prepared for an exploratory laparotomy. Four liters of dark red bloody ascites was evacuated. The uterus was in normal size. Both ovaries were adherent to the surrounding tissue and replaced by complex, solid masses with a maximum diameter of 20 cm and 15 cm, respectively. There were many nodosity (0.1-5 cm in diameter) excrescences in the pelvic wall, as well as mesentery, the serosal surface of the intestines and omentum. The liver and spleen were normal. Frozen section of the both ovary masses demonstrated a malignant ovarian stromal tumor. A cytoreductive surgery was performed consisting of a total abdominal hysterectomy, bilateral salpingo-oophorectomy, partial omentectomy, peritoneal metastases resection. The residual tumors are less than 2 cm in diameter and the stage classification after the surgery was FIGO IIIc.

**Figure 3:**
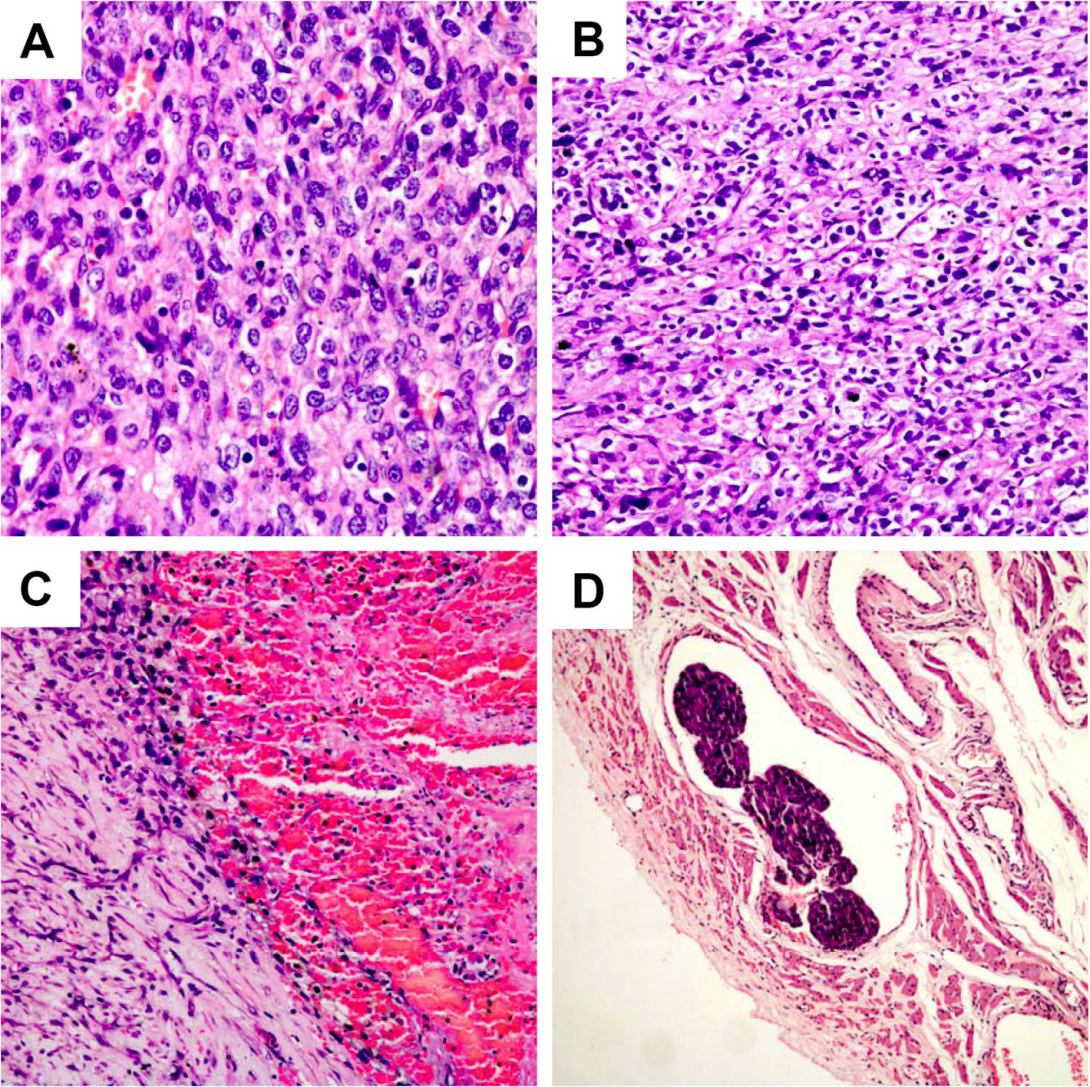
**Malignant Steroid cell tumor, NOS.** Microscopic appearance: Cells with irregular nuclei or atypia **(A**, H&E, 40×), and mitosis **(B**, H&E, 20×) are arranged in a diffuse pattern of columns or nests separated by a rich vascular network. Diffuse hemorrhage and necrosis are observed **(C**, H&E, 20×). The presence of intravascular tumor embolus further supports the diagnosis of malignancy **(D**, H&E, 10×).

The cut surface of the specimen showed a solid mass including serous, myxoid, hemorrhagic, and necrotic contents with calcific deposits. The peripheral nodular wall was composed of yellowish solid tissue. Microscopically, diffusely arranged tumor cells with irregular nuclei or atypia were identified (Figure 3A). More than 10 mitotic figures per 10 high-power fields were seen (Figure 3B). Diffuse hemorrhage and necrosis are observed (Figure 3C. The presence of intravascular tumor embolus further supports the diagnosis of malignancy (Figure 3D). No crystals of Reinke were identified. Immunohistochemistry revealed a result of CKpan (-), Vimentin (+), EMA (-), ER (-), PR (-), CD99 (+), Calretinin (++), Inhibin-α (++), ki-67 (-), P53 (-), S-100 (-), Alpha-Fetoprotein (AFP) (-),CD68 (+), Leucocyte common antigen (LCA) (+), Melanoma marker (HMB45) (+). These findings indicated a malignant ovarian steroid cell tumor, NOS.

Her serum total testosterone fell down significantly one week after surgery, but it had been rising continuously since then (Figure 2 and Table 1). Although the patient was given 4 cycles of standard PVB (cisplatin, vincristine and bleomycin, every three weeks) chemotherapy, she died of tumor recurrence in pelvic and abdominal cavity 10 months after operation.

## Discussion

Steroid cell tumors are defined as ovarian neoplasms composed of steroid hormone-secreting cells. They have been divided into three subtypes according to their cell of origin: stromal luteoma arising from ovarian stroma, Leydig cell tumor arising from Leydig cells in the hilus, and steroid cell tumor not otherwise specified (NOS) when the lineage of the tumor is unknown [1]. The last type accounts for approximately 60% of steroid cell tumors, 25%–45% of which are clinically malignant [12].

Steroid cell tumors, NOS, usually occur in adults with an average age at diagnosis of 47 years [13]. The clinical presentations are not specific, including abdominal pain, distention, and bloating. However, the more significant presentations are those associated with the hormonal activity and virilizing properties of the tumor, which accounts for 56%-77% of patients [7]. Signs and symptoms of masculinizing tumors usually take place in two definite phases, an early phase of defeminization and a subsequent phase of masculinization. Typically, a menstruating female will first notice oligomenorrhea or amenorrhea, which occurred in both of our patients. Common signs of masculinization include hirsutism, acne, clitoral enlargement, increased libido, sterility, enlargement of the larynx, deepening of the voice, and temporal alopecia.

The diagnosis of steroid cell tumors, NOS, should be made on the basis of the clinical virilizing syndromes, the microscopic pictures, as well as immune reactivity to some immunohistochemical markers. Inhibin and calretinin were thought to be sensitive and robust markers in differentiating sex cord-stromal from non-sex cord-stromal tumors [14]. Our patients had a positive inhibin and calretinin stain, which supported the diagnosis of steroid cell tumors, NOS.

The most important factor to be determined in steroid cell tumors of the ovary is whether the tumor has malignant features or not. The incidence of clinical malignancy among the steroid cell tumors has been documented. Hayes and Scully identified five pathologic features that are highly associated with malignancy [1] (Table 2). We found that our second patient met all of the five malignant features (Table 2). In addition, we identified the intravascular tumor embolus in our malignant case. The presence of tumor embolus not only supports the diagnosis of malignancy, but also predicts a poor prognosis of such disease.

**Table 2:** Predictive pathological characteristics of malignancy for ovarian steroid cell tumors, NOS (by Hayes and Scully)

**Pathologic characteristics**	**Percentage associated with the malignancy (%)**	**Our cases**	
		**Case 1**	**Case 2**
Tumor diameter > 7 cm	78	No	Yes
Mitotic figures per 10 high-power fields ≥ 2	92	No	Yes
Necrosis	86	No	Yes
Hemorrhage	77	No	Yes
Grade 2 or 3 nuclear atypia	64	No	Yes

The mainstay treatment of ovarian steroid cell tumor is surgery. In general, conservative surgery with unilateral oophorectomy and proper staging should be performed in women with stage I disease who desire future fertility. For women who have completed childbearing, total abdominal hysterectomy with bilateral salpingo-oophorectomy and complete surgical staging is indicated [15]. Base on the literature study, we believe that our benign case is the first one that underwent a laproscopic cystectomy. Adjuvant chemotherapy should be based on the histologic appearance of the tumor and on its surgical stage. However, there are no well defined chemotherapy guidelines for clinical management of steroid cell tumor. PVB (cisplatin, vincristine, and bleomycin) or BEP (bleomycin, etoposide, and cisplatin) was recommended by some authors [15].

## Conclusions

Steroid cell tumors, NOS, are rare ovarian tumors which can be difficult to diagnose. Careful history and physical examination, in addition to laboratory values and imaging studies, are helpful in making the diagnosis. These tumors should be considered a cause of isosexual precocious puberty in children and virilization in adults. Any patient who presents with virilism should be investigated systematically to determine if the high testosterone levels are of an adrenal or ovarian origin. Therapy should be individualized based on tumor histology, surgical staging, and the desire for fertility preserving. Malignant steroid cell tumors, NOS, should be managed with surgical removal followed by combination chemotherapy.

## Competing interests

The authors declare that they have no competing interests.

## Authors’ contributions

WJ and XT drafted the manuscript. FF participated in the collecting of patients’ laboratory data. SFZ and CJX are involved in design, acquisition, interpretation and manuscript preparation. All authors had read and approved the final manuscript.
